# The role of microRNAs in the modulation of cancer-associated fibroblasts activity during pancreatic cancer pathogenesis

**DOI:** 10.1007/s13105-022-00899-0

**Published:** 2022-06-29

**Authors:** Lawrence N. Barrera, P. Matthew Ridley, Camino Bermejo-Rodriguez, Eithne Costello, Pedro A. Perez-Mancera

**Affiliations:** 1 Department of Molecular and Clinical Cancer Medicine, University of Liverpool, Liverpool, UK; 2 Department of Molecular Cell Biology, School of Medicine, Faculty of Clinical and Biomedical Sciences, University of Central Lancashire, Preston, PR1 1JQ UK

**Keywords:** microRNAs, Cancer-associated fibroblasts (CAFs), Pancreatic cancer, Tumour pathogenesis, Response to therapy

## Abstract

Pancreatic ductal adenocarcinoma (PDAC) is the deadliest of the common cancers. A major hallmark of PDAC is an abundant and dense fibrotic stroma, the result of a disproportionate deposition of extracellular matrix (ECM) proteins. Cancer-associated fibroblasts (CAFs) are the main mediators of PDAC desmoplasia. CAFs represent a heterogenous group of activated fibroblasts with different origins and activation mechanisms. microRNAs (miRNAs) are small non-coding RNAs with critical activity during tumour development and resistance to chemotherapy. Increasing evidence has revealed that miRNAs play a relevant role in the differentiation of normal fibroblasts into CAFs in PDAC. In this review, we discuss recent findings on the role of miRNAs in the activation of CAFs during the progression of PDAC and its response to therapy, as well as the potential role that PDAC-derived exosomal miRNAs may play in the activation of hepatic stellate cells (HSCs) and formation of liver metastasis. Since targeting of CAF activation may be a viable strategy for PDAC therapy, and miRNAs have emerged as potential therapeutic targets, understanding the biology underpinning miRNA-mediated tumour cell-CAF interactions is an important component in guiding rational approaches to treating this deadly disease.

## Introduction

Pancreatic ductal adenocarcinoma (PDAC) is the fourth leading cause of cancer-related mortality worldwide and is expected to become second by 2030 [96]. Late diagnosis of patients when tumours are at advanced stages and a strong resistance to systemic chemotherapy and radiotherapy are the major reasons for this dire clinical outcome (5-year survival rate of ~ 9%) [59, 96, 110]. It is well established that activating mutations in KRAS (detected in over 90% of PDAC cases) lead to the development of low-grade preinvasive lesions (preinvasive pancreatic intraepithelial neoplasia; PanIN) that progress to high-grade PanIN lesions and eventually PDAC following the acquisition of additional genetic alterations that frequently involve inactivation of the tumour suppressor genes *TP53*, *P16/CDKN2A* and *DPC4/SMAD4* [47, 91].

PDAC is characterised by the development of complex and unique histological features. The bulk of the tumour comprises actively proliferating neoplastic cells and a desmoplastic stroma that accounts for up to 90% of the tumour volume. The stroma consists of activated cancer-associated fibroblasts (CAFs), immune cells, and endothelial cells embedded in a dense extracellular matrix (ECM) composed of proteins, glycoproteins, and proteoglycans [32, 79]. This reactive stroma provides an exclusive tumour microenvironment (TME) that has been demonstrated to drive tumour progression, dissemination, and resistance to therapy [53, 83, 92]. Intriguingly, studies have shown that the PDAC microenvironment also harbours tumour suppressive properties [18, 20, 86, 97, 99]. These apparently opposing activities emphasise the complex role that the TME plays in PDAC development, underpinning the need of a better comprehension of the stromal biology, as well as the molecular and cellular mechanisms that operate and control the crosstalk between neoplastic cells and components of the TME during tumour progression. Expanding our knowledge in this area will contribute to improved therapeutic intervention strategies to fight this disease.

CAFs are the most abundant component of the pancreatic TME and play an essential role during the pathogenesis of PDAC, including tumour development and response to therapy [12]. Secretion of components of the ECM, chemokines, and cytokines by CAFs is considered to be the most significant contributor to pancreatic cancer desmoplasia [12, 55, 82, 103]. However, despite recent advances in understanding the biology of CAFs, our knowledge of the crosstalk between tumour cells and CAFs remains inadequate, which has contributed to the inefficient design of therapeutic approaches to tackle pancreatic cancer.

MicroRNAs (miRNAs) are highly conserved small non-coding RNA molecules (~ 22 nucleotides in length) that negatively regulate post-transcriptional gene expression. Mechanistically, miRNAs bind to the 3’-untranslated region of messenger RNAs (mRNAs) and silence gene expression through degradation or translational inhibition of target mRNAs. miRNAs have been shown to have both tumour suppressive and promoting activities depending on the cellular and molecular context [51, 90, 102] and, importantly, accumulating evidence indicates that miRNAs play a critical role in remodelling the tumour microenvironment [87, 101, 113]. In this review, we discuss progress in understanding the roles of miRNAs in the regulation of CAF activation during pancreatic cancer development and response to therapy.

## Origin of CAFs

Despite huge efforts to elucidate where CAFs come from, their origin remains controversial, and the identification of cellular sources of CAFs persists as a significant unresolved challenge. This is largely due to the difficulty in categorising and defining distinct CAF populations [12, 55, 82, 103]. Two main factors have contributed to our failure to accurately delineate CAF populations. Firstly, most of the studies with CAFs have been performed using in vitro approaches, which are unable to faithfully recapitulate the physiology of the in vivo microenvironment. Secondly, the lack of relevant in vivo lineage tracing mouse models, due to the unavailability of CAF-specific molecular markers, has hampered our ability to unravel CAF biology [103]. Indeed, in vivo approaches typically involve the use of transgenic or knock-in alleles to express *CRE* recombinase in restricted cellular compartments, where CRE drives the expression of reporter genes (usually fluorescent proteins) that label and track specific cell populations during the whole life of the animal [1]. However, the lack of discernible CAF markers has precluded the selection of *CRE*-driving promoters to tag and trace specific CAF populations in vivo.

**Fig. 1:**
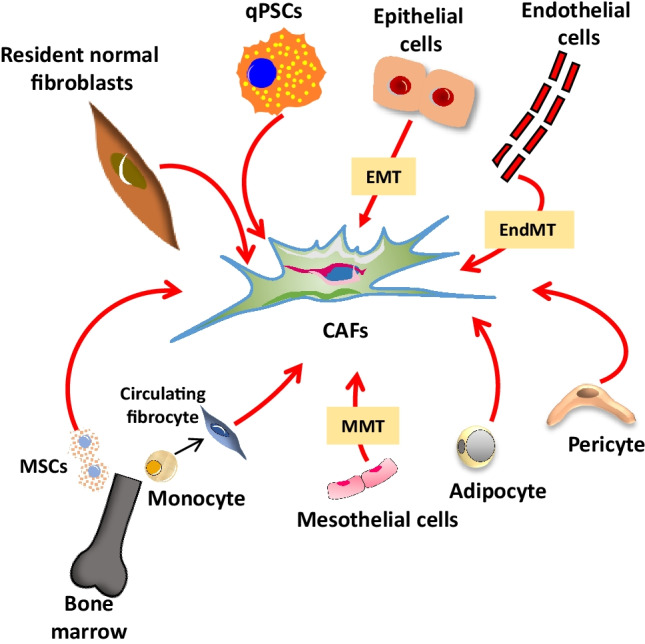
Origin of cancer-associated fibroblasts (CAFs) in PDAC. Schematic illustration of the cellular sources of CAFs. Cell types include resident normal fibroblasts, quiescent pancreatic stellate cells (qPSCs), epithelial cells, endothelial cells, pericytes, adipocytes, mesothelial, bone marrow–derived fibrocytes, and mesenchymal stem cells (MSCs). *EMT*, epithelial-to-mesenchymal transition; *EndMT*, endothelial-to-mesenchymal transition; *MMT*, mesothelial-to-mesenchymal transition

Multiple lines of evidence have suggested that CAFs mainly derive from local precursors, including quiescent resident fibroblasts, hepatic stellate cells (HSCs), and pancreatic stellate cells (PSCs) [3, 55, 84, 126] (Fig. 1). Recently, an elegant study employing a dual reporter mouse model provided support for this concept. Arina and colleagues found that CAFs expressing collagen type I alpha 1 (Col1a1) and alpha-smooth muscle actin (αSMA), two CAF-associated markers, originated mainly from precursors located in the tumour microenvironment, while the contribution from bone marrow circulating precursors was infrequent [4]. In the context of pancreatic cancer, the most important sources of CAFs are PSCs. Activation and transformation of quiescent PSCs into active CAFs are driven by different mechanisms, including cytokines and growth factors such as TGF-β, IL-1, IL-10, PDGF, and CTGF [7, 11, 72, 105], exosomes-released miRNAs [70], oxidative stress [64], and epigenetic mechanisms [10].

Interestingly, bone marrow–derived mesenchymal stem/progenitor cells (MSCs) have been observed in the peripheral blood of patients with PDAC [112], and MSCs incubated with conditioned media from the human pancreatic cell line PANC1 were found to upregulate the expression of CAF-associated genes including *αSMA* [75]. Collectively, these findings support the notion that circulating MSC precursors may also contribute to the population of CAFs in pancreatic cancer [4]. Additional studies have reported that CAFs can also derive from the differentiation of other cell types. Fibrocytes are circulating mesenchymal cells derived from monocyte precursors that can be recruited to the neoplastic tissue and differentiate into CAFs [23, 98]. Other cell types from mesenchymal origin that have been shown to give rise to CAFs include adipocytes [13, 56] and pericytes [44]. Furthermore, transdifferentiation from mesothelial cells through mesothelial-to-mesenchymal (MMT) transition [104], endothelial cells through endothelial-to-mesenchymal (EndMT) transition [128], and even epithelial cells through epithelial-to-mesenchymal (EMT) transition [52] have also been reported to be potential precursors of CAF populations (Fig. 1).

Taken together, these studies confirm that CAFs constitute a highly heterogenous cell population with multiple origins and routes of activation. The identification of specific molecular markers to definitively categorise different CAF populations is essential to advance our knowledge of CAF biology.

## Fibroblast heterogeneity in PDAC

Fibroblasts were originally treated as a homogeneous cell population present in a quiescent state or as activated CAFs. In pancreatic tissue, quiescent PSCs exhibit cytoplasmic deposits of vitamin A-lipid droplets that inhibit PSC activation. Once stimulated, PSCs lose the ability to store vitamin A and acquire an activated phenotype that is characterised by increased proliferation and secretion of components of the ECM that intensify tumour desmoplasia [71]. However, increasing experimental evidence has demonstrated that fibroblast activation in physiological contexts is more complex. The rapid development of next-generation sequencing technologies is shedding new light on the existence of diverse CAF subtypes, with different activities, within the tumour microenvironment [21, 29, 35, 57, 63, 65, 94, 119].

**Fig. 2:**
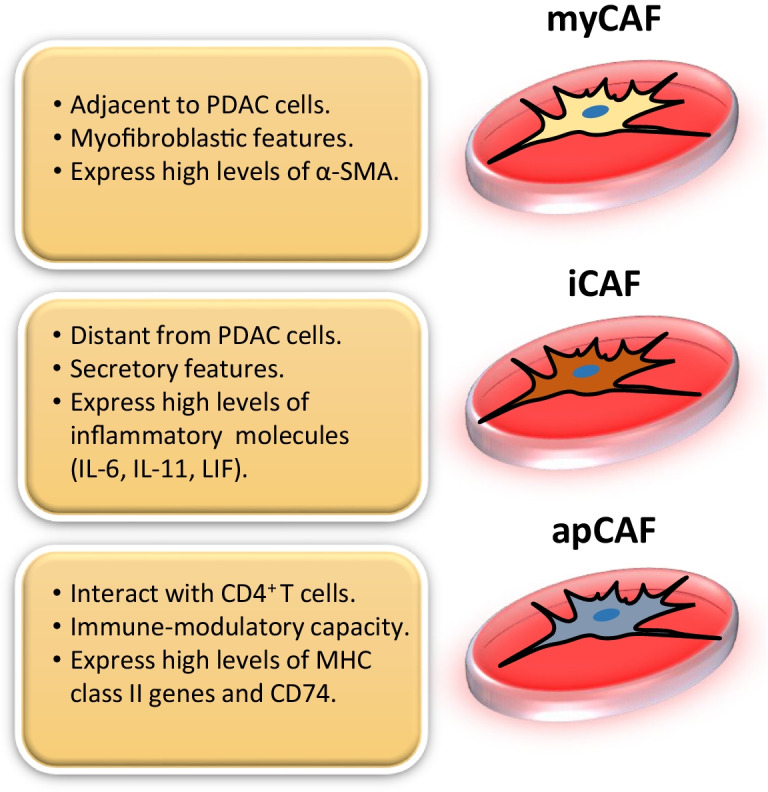
CAF subtypes in PDAC. Three distinct cancer-associated fibroblast (CAF) subpopulations have been identified and characterised in PDAC: myofibroblastic CAF (myCAF), inflammatory CAF (iCAF), and antigen-presenting CAF (apCAF)

In the case of PDAC, Ӧhlund and colleagues identified the coexistence of two subtypes of CAFs. Employing an innovative three-dimensional (3D) co-culture approach of murine wild-type PSCs and PDAC organoids derived from *Kras*^*LSL−G12D/*+^, *Trp53*^*LSL−R172H/*+^, *Pdx1*^*Cre/*+^ (KPC) mice, it was found that PSCs differentiated into two spatially separated and reversible CAF subpopulations. The first subpopulation, termed inflammatory CAF (iCAF), was activated by paracrine factors produced by neoplastic cells. iCAFs localised distantly from cancer cells and were characterised by lower expression of αSMA and high level of inflammatory mediators such as interleukin-6 (IL-6), IL-11, and leukaemia inhibitory factor (LIF) (Fig. 2). The second subtype, named myofibroblastic CAF (myCAF), was activated by juxtracrine interaction with tumour cells and had periglandular location. myCAFs had a myofibroblastic phenotype characterised by high levels of αSMA expression and reduced secretion of inflammatory cytokines (Fig. 2). Interestingly, conversion from one CAF subtype to another was highly dynamic, depending on molecular cues in the tumour microenvironment [81]. Subsequent studies have provided new insights into the signalling pathways governing the activation and regulation of iCAFs and myCAFs. It has been reported that TGF-β secreted by both neoplastic and regulatory T (Treg) cells promotes myCAF differentiation, while causing attenuation of the iCAF phenotype by downregulating IL1R1 expression [11, 25, 131]. On the other hand, secretion of IL1 by pancreatic tumour cells mediates activation of the NF-κB and JAK/STAT signalling pathways, promoting an iCAF phenotype and the secretion of tumour-promoting cytokines and chemokines such as IL-6, LIF, and CXCL1 [11, 111].

Additional studies have confirmed the heterogeneity of CAFs in PDAC at single-cell resolution. Employing single-cell RNA sequencing of human and mouse PDACs, Elyada and colleagues verified the existence of myCAFs and iCAFs associated with unique gene signatures. Importantly, this study identified a novel CAF subpopulation, dubbed antigen-presenting CAF (apCAF), which expresses MHC class II-related genes and *CD74*, and have an immune-modulatory role through activation of CD4 + T cells [29] (Fig. 2). Interestingly, this apCAF subpopulation has a similar transcriptional signature to mesothelial cells [25] that have been found to be potential CAF precursors [104]. Further single-cell transcriptomic approaches have demonstrated that while myCAFs were abundant in preinvasive intraductal papillary mucinous neoplasm (IPMN) lesions, iCAFs were only detected in invasive PDAC specimens. These findings strongly suggest that transdifferentiation of fibroblast/PSCs into myCAFs happens in early stages of pancreatic cancer development [9]. An additional study has provided further insight into the origin of myCAFs. Using an in vivo lineage-tracing approach, Garcia and colleagues revealed that two subpopulations of Gli1^+^- and Hoxb6^+^-expressing fibroblasts coexist at similar levels in healthy pancreas. However, only Gli1^+^ fibroblasts expand in preinvasive PanIN lesions and contribute to the tumour stroma during PDAC development. Interestingly, Gli1^+^ fibroblasts express αSMA and may represent early precursors of the myCAF population [34]. Finally, a study which employed mass cytometry to illustrate the stromal composition of diverse normal and neoplastic murine tissues [50] found that while CD105-positive pancreatic fibroblasts do not affect tumour growth in vivo, CD105-negative fibroblasts confer a strong tumour-suppressive activity.

Taken together, these studies demonstrate that CAFs have distinct functional roles during the progression from preinvasive lesions to invasive of PDAC.

## Fibroblast heterogeneity in different pancreatic diseases

Both pancreatic neoplastic (PDAC) and inflammatory (chronic pancreatitis, CP) tissues display a compact desmoplasia comprising abundant ECM and expansion of stromal cell populations. Although PSCs derived from PDAC and CP share cellular and functional features, specific and differential transcriptional profiles have been associated with each cell population. For example, cadherin EGF LAG seven-pass G-type receptor 3 (*CELSR3*) was found to be upregulated in PDAC-associated PSCs compared to PSCs derived from CP, while Pre-B-cell leukaemia transcription factor 1 (*PBX1*) was expressed at higher levels in PSCs present in inflammatory conditions (CP) compared to PDAC-derived PSCs [30]. A better understanding of the molecular basis governing the activation of fibroblast/PSCs in different pancreatic contexts will provide insight into the molecular basis of CAF activation.

**Fig. 3:**
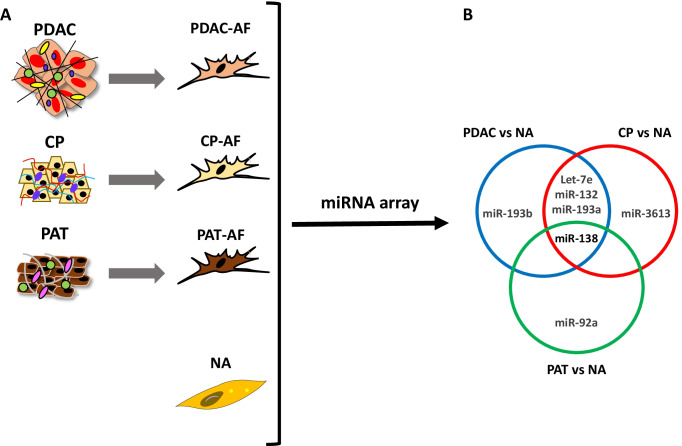
microRNA expression in pancreatic disease-associated fibroblasts. **A** miRNA arrays were employed to identify miRNA expression profiles in activated fibroblasts isolated from pancreatic ductal adenocarcinoma (PDAC), chronic pancreatitis (CP), periampullary tumour (PAT), and adjacent normal (NA) tissues. **B** Venn diagram depicting differentially expressed miRNAs between disease-associated fibroblasts (PDAC, CP, PAT) versus normal-activated (NA) fibroblasts

Recently, we employed a comprehensive approach to evaluate gene expression patterns of primary activated pancreatic fibroblasts isolated from a variety of pancreatic disease types including PDAC, CP, and periampullary tumours (PATs). The analysis of miRNA and gene expression profiles revealed significant overlap in the genetic signatures of disease-derived fibroblasts compared to normal activated (NA) fibroblasts. Indeed, only 6.6% and 7.3% of genes were transcriptionally altered in CP-derived activated fibroblasts (CP-AFs) versus NA fibroblasts, and PAT-derived activated fibroblasts (PAT-AFs) versus NA fibroblasts, respectively. Moreover, this analysis revealed that around 32% of genes were differentially expressed between PDAC-derived activated fibroblasts (PDAC-AFs) and NA fibroblasts [8]. These findings suggest that the differential regulation of a restricted subset of genes governs the functional heterogeneity of fibroblasts between pancreatic diseases. When comparisons were circumscribed to PDAC-AFs and CP-AFs, it was observed that Tenascin C (TNC), an ECM glycoprotein with therapeutic relevance [73], was found to be upregulated in PDAC-AFs compared to CP-AFs. Consistent with this, immunohistochemical analysis found that TNC was upregulated in the stromal compartment of PDAC relative to CP and, importantly, circulating TNC was significantly upregulated in serum of PDAC patients compared with CP patients. Collectively, these findings support the clinical relevance of TNC as a disease biomarker. Mechanistically, miR-137, reported to be downregulated in PDAC [78, 123], was linked to the increased TNC expression found in PDAC-associated fibroblasts [8], supporting the important role for miRNAs in the activity of CAFs. Interestingly, our miRNA-profiling analysis unveiled a subset of five miRNAs dysregulated in PDAC-AFs compared with NA fibroblasts (let-7e, miR-132, miR-193a, miR-193b, and miR-138), with three of them (let-7e, miR-132, and miR-193a) also dysregulated between CP-AFs and NA fibroblasts, and miR-138 dysregulated in both CP-AFs and PAT-AFs. Additionally, miR-3613 was only found dysregulated in CP-AFs, while miR-92a dysregulation was exclusively altered in PAT-AFs (Fig. 3).

Collectively, this study indicates that pancreatic-disease fibroblasts have highly conserved transcriptome and miRNA profiles, and a subset of genes and miRNAs differentially regulated by the surrounding pancreatic tissue seems to drive the reprogramming of normal fibroblasts into PDAC-AFs, CP-Afs, or PAT-AFs.

## Role of miRNAs in the activation of PDAC-associated CAFs

Early miRNA profiling analysis showed that miRNAs were differentially expressed in human neoplasias, including PDAC [15, 66]. Additional studies revealed that miRNAs played an important role during tumour pathogenesis and could be used as biomarkers for tumour diagnosis [38, 115]. Importantly, miRNAs have been found to regulate the transition from quiescent resident fibroblasts into CAFs in the tumour microenvironment, modulating tumour development [76, 107, 121, 125]. In the pancreatic context, early observations showed that the miRNA expression profile was altered during the activation of rat PSCs in cell culture. Indeed, Masamune and colleagues employed miRNA microarrays in quiescent (day 1) and culture-activated (day 14) rat PSCs and found 84 miRNAs differentially expressed (42 miRNAs were upregulated and 42 downregulated) in activated PSCs compared with quiescent counterparts [69]. Subsequent studies have confirmed that miRNA dysregulation plays a relevant role in CAF activation, tumour cell-CAF interactions, and PDAC development.

**Fig. 4:**
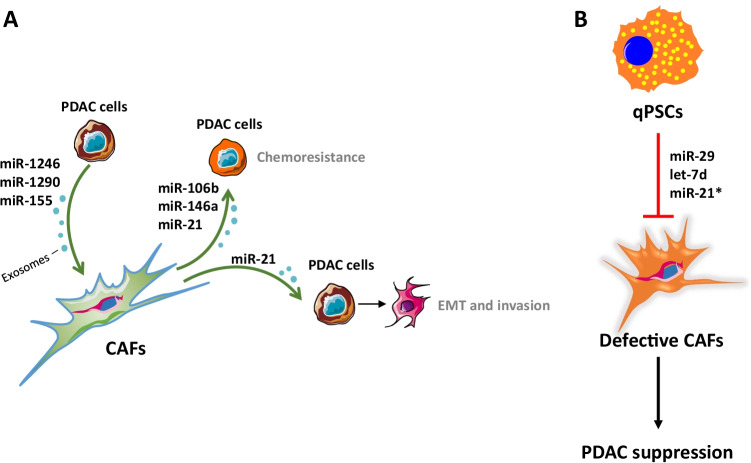
microRNAs modulate PDAC pathogenesis. Examples of miRNAs with **A** tumour-promoting and **B** tumour-suppressive activities in PDAC. (*) In some contexts, miR-21 has been shown to act as a tumour suppressor. Blue circles represent exosomes. *qPSC*, quiescent pancreatic stellate cells; *CAFs*, cancer-associates fibroblasts; *PDAC*, pancreatic ductal adenocarcinoma; *EMT*, epithelial-to-mesenchymal transition

Exosomes are extracellular vesicles that have be shown to transfer mRNAs and miRNAs between cells [118]. During the last few years, diverse studies have revealed that exosomal miRNAs play an important role in the crosstalk between pancreatic neoplastic cells and CAFs (Fig. 4A) [108]. Pang and colleagues showed that normal pancreatic fibroblasts derived from wild-type mice differentiated into CAF-like cells after being incubated with PDAC cell-derived microvesicles containing miR-155. In this model, activation of normal fibroblasts may be mediated, at least partially, by miR-155-mediated targeting of the pro-apoptotic modulator TP53INP1 [88]. Moreover, PDAC cell exosome-mediated transference of miR-1246 and miR-1290 was found to promote activation of primary human PSCs, denoted by the upregulation of procollagen type I C-peptide (*PIP*) and *αSMA* [70]. Interestingly, CAF-derived exosomal miRNAs have been demonstrated to play a relevant role in chemoresistance. Fang and colleagues reported that gemcitabine-elevated miR-106b expression in PDAC-associated CAFs, and exosome-mediated transference of miR-106b from CAFs to pancreatic tumour cells increased resistance to gemcitabine by a mechanism involving miR-106b-mediated targeting of TP53INP1 [31]. An independent study showed that gemcitabine upregulated miR-146a and Snail in pancreatic CAFs, and exosomes secreted by gemcitabine-treated CAFs increased both miR-146a and Snail levels in PDAC cells in culture, increasing chemoresistance [100]. These studies provide a rationale for the potential use of miR-106b and miR-146a as therapeutic targets for patient treatment stratification in PDAC.

One of the most commonly upregulated miRNAs in human neoplasias, miR-21, has been shown to play an important role in the crosstalk between tumour cells and CAFs [16, 33, 36, 60, 85, 109, 120]. Interestingly, this crosstalk can be mediated by exosomal miR-21 released by CAFs [6, 27]. In the pancreatic cancer context, miR-21 is upregulated in CAFs compared to normal PSCs [2], and numerous studies have confirmed that CAF-derived miR-21 supports PDAC dissemination. Kadera and colleagues showed that PDAC cells stimulated miR-21 upregulation in CAFs, and high miR-21 expression in the tumour stroma was associated with tumour invasion, lymph node metastasis, and shorter survival. Interestingly, miR-21 expression was stronger around neoplastic ducts and it decreased laterally in a circular gradient away from tumour cells, indicating that crosstalk signalling between pancreatic tumour cells and CAFs regulates miR-21 expression [54]. Additional studies have reinforced the functional relevance of CAF-derived miR-21 in the modulation of the invasive behaviour of PDAC cells. Thus, Chen and colleagues found that miR-21 inhibition reduced glycolysis in CAFs, and this was associated with decreased invasiveness of pancreatic tumour cells [17]. Furthermore, it has been observed that exosome-mediated transfer of miR-21 from CAFs to pancreatic neoplastic cells induces EMT, activation of KRAS downstream effector pathways, and migration of PDAC cells [67]. An independent study has confirmed the oncogenic role of miR-21 in pancreatic cancer. Chu and colleagues found that miR-21 inhibition in PDAC cells derived from *Kras*^*LSL−G12D/*+^, *Trp53*^*LSL−R172H/*+^, *Pdx1*^*Cre/*+^ (KPC) mice reduced proliferation, migration, and invasion associated with the downregulation of Kras effector pathways [19]. Interestingly, this study has also revealed that miR-224 is specifically upregulated in PDAC CAFs, and ectopic expression of miR-224 promotes pancreatic normal fibroblast activation [19].

Importantly, miR-21 has also been reported to have therapeutic relevance. Indeed, diverse studies have demonstrated that high levels of miR-21 increase resistance to gemcitabine in PDAC cell cultures and, in the clinical setting, miR-21 expression correlated with poor prognosis in patients treated with gemcitabine [36, 77, 89]. Zhang and colleagues revealed that miR-21 expression in human PDAC tissue was associated with an elevated number of CAFs and a poorer response to gemcitabine. Notably, gemcitabine increased miR-21 expression in CAFs, but not in neoplastic cells. Moreover, ectopic miR-21 expression in CAFs increased invasiveness of PDAC cells in culture and promoted resistance to gemcitabine in a xenograft mouse model. In this context, miR-21-mediated CAF activation, denoted by increased secretion of metalloproteases, cytokines, and growth factors, was driven by miR-21-mediated PDCD4 targeting. Collectively, these findings strongly support the notion that miR-21 induces resistance to gemcitabine by regulating CAF activity [130]. However, an independent study showed that expression levels of miR-21 in CAFs correlated with decreased survival in PDAC patients who received 5-FU but not gemcitabine [26]. Further studies will be needed to clarify the therapeutic relevance of miR-21 in PDAC.

Conversely, research using a mouse model of PDAC has shown that, in some contexts, miR-21 may have tumour suppressor activities. Schipper and colleagues observed that pancreatic expression of Kras^G12D^ and concomitant p53 deficiency cooperated with systemic ablation of miR-21 to accelerate pancreatic cancer development in a well-established mouse model of PDAC (*Kras*^*LSL−G12D/*+^, *Trp53*^*LoxP/*+^, *Pdx1*^*Cre/*+^, *Mir-21*^*KO/KO*^ (KP^F/+^C21KO)) [106]. Remarkably, KP^F/+^C21KO mice developed preinvasive mucinous cystic neoplasia (MCN) lesions that progressed to invasive PDAC with complete penetrance. KP^F/+^C21KO mice showed reduced overall survival (12.5 weeks) compared with KP^F/+^C counterparts (23 weeks). Analysis of KP^F/+^C21KO tumours showed that miR-21 ablation drastically reduced CAF activation, denoted by α-SMA expression, while angiogenesis and immune infiltration were increased [106]. Although these findings seem to contradict the generalised view of miR-21 as an oncogenic miRNA [33], additional studies employing a miR-21 conditional knock-out strain will be necessary to exclude potential adverse effects of the germline inactivation of miR-21 in the activation of fibroblasts. Furthermore, the phenotype of the KP^F/+^C21KO mice supports the notion that a dense desmoplastic stroma can restrain rather than stimulate PDAC development [18, 20, 86, 97, 99]. Importantly, this work has major clinical implications, as therapeutic approaches aiming at modulating miRNA activity in tumours should consider the effect of modulating the target miRNA expression in both the neoplastic and stromal compartments.

Additional experimental evidence has unveiled other CAF-derived miRNAs with tumour suppressor activities in PDAC (Fig. 4B). Kwon and colleagues showed that TGFβ downregulated miR-29 expression in mouse PSCs (mPSCs), human PSCs (hPSCs), and human PDAC-derived CAFs in culture. Moreover, miR-29 knockdown increased expression of ECM proteins in mPSCs and hPSCs, and ectopic miR-29 in hPSCs decreased viability and colony formation of human PDAC cells in co-culture. Importantly, miR-29 was found to be downregulated in a mouse model of PDAC (*Kras*^*LSL−G12D/*+^, *Pdx1*^*Cre/*+^) and in human PDAC [61]. An additional study proved that ectopic expression of the miRNA let-7d suppressed activation of human PSCs, decreasing the expression of the fibrosis markers *αSMA* and *COL1A1* by targeting Thrombospondin 1 (*THBS1*) [5]. Interestingly, the same group found that inhibition of let-7d in human PSCs promoted TGFβ-mediated *αSMA* and *COL1A1* expression, and reduced serum let-7d levels correlated with shorter survival in PDAC patients treated with gemcitabine-based chemotherapy [114], confirming that let-7d can be used as a biomarker of therapeutic response.

Collectively, the studies presented above highlight the central role of miRNAs in the activation of CAFs and in the pathogenesis of PDAC, opening new avenues for the design of therapeutic intervention strategies.

## PDAC-derived exosomal miRNAs as potential drivers of liver metastasis

A high dissemination potential and the formation of distant metastasis are major hallmarks of PDAC [46, 59]. The liver is the most common metastatic site, with hepatic metastatic deposits found in up to 76% of patients with PDAC [124]. Hepatic stellate cells (HSCs) are considered to be the hepatic counterparts of PSCs, and activated HSCs transdifferentiate into collagen-producing myofibroblasts that stimulate the generation of a fibrotic microenvironment supporting the formation of metastasis in the liver [116]. Multiple molecules and signalling pathways are involved in the activation of HSCs, including growth factors, cytokines, adipokines, Hedgehog ligands, and miRNAs [116]. Significantly, primary tumour-derived factors have been demonstrated to activate HSCs to drive the formation of a hepatic metastatic niche [24, 28, 74]. For example, it was shown that the secretion of tissue inhibitor of metalloproteinases-1 (TIMP1) by pancreatic premalignant lesions caused the activation of HSCs via CD63 signalling, creating a premetastatic niche in the liver [39]. Moreover, PDAC-derived exosomes expressing macrophage migration inhibitory factor (MIF) were found to induce the secretion of TGFβ by Kupffer cells (KCs) in the liver. Subsequently, KC-derived TGFβ activated HSCs that secreted fibronectin which, in turn, recruited bone marrow-derived cells preparing the metastatic niche in the liver [22]. The same group showed that exosomes derived from human and mouse pancreatic cancer cells were particularly taken up by liver and, remarkably, this was mediated by the exosomal integrin α_v_ (ITGα_v_). Importantly, exosomal ITGα_v_ levels in pancreatic cancer patient plasma correlated with a higher liver metastasis burden [45]. An additional study showed that PDAC cells recruited metastasis-associated macrophages (MAMs) to the liver. MAMs secreted granulin, inducing the transdifferentiation of HSCs into myofibroblasts that, in turn, secreted components of the ECM, including periostin, which supported the colonisation of PDAC cells in the liver and the formation of metastatic deposits [80].

**Fig. 5:**
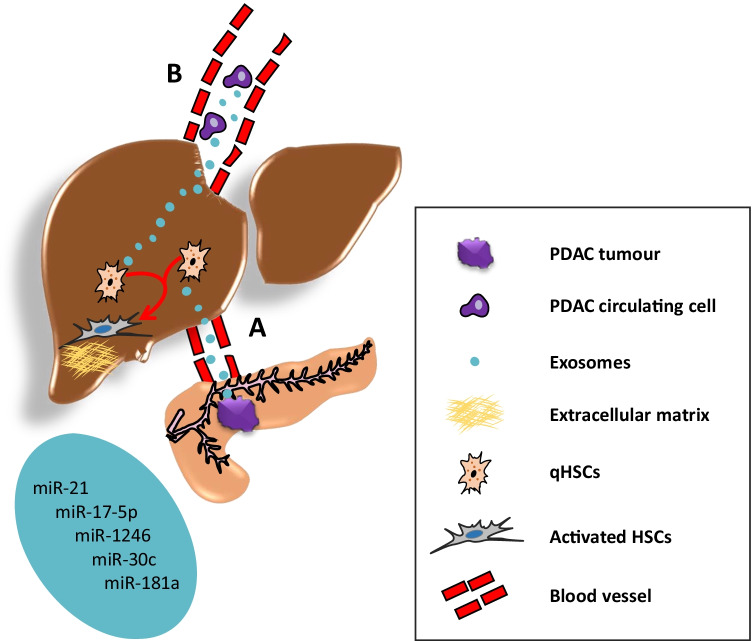
Exosomal miRNAs promote activation of quiescent hepatic stellate cells (qHSCs). Exosomes derived from the primary PDAC tumour cells (**A**) or PDAC circulating tumour cells (**B**) deliver to the liver miRNAs that activate qHSCs and potentially mediate the formation of a hepatic pre-metastatic niche

Importantly, exosome-derived miRNAs have been shown to trigger stroma remodelling and formation of the hepatic metastatic niche. Hsu and colleagues showed that bone marrow–derived exosomes containing miR-92a enhanced HSC activation and ECM deposition that contributed to the formation of a hepatic pre-metastatic niche in a mouse model of lung cancer [48]. These findings strongly support the notion that exosomal miRNAs derived from PDAC primary tumours, or from PDAC circulating cells, may have a significant role in the activation of HSCs to support the growth and colonisation of PDAC cancer cells in the hepatic metastatic microenvironment (Fig. 5). Interestingly, several miRNAs involved in the activation of HSCs have been found upregulated in circulating exosomes of patients with pancreatic cancer. For instance, miR-21 was found to be highly upregulated in activated HSCs [129, 132], and it has been shown to enhance the transdifferentiation of HSCs into CAFs [133]. Notably, exosomal miR-21 has been found to be significantly upregulated in plasma and serum of patients with pancreatic cancer compared with healthy controls [37, 62, 93]. Additional miRNAs upregulated in circulating exosomes of pancreatic cancer patients which induce HSC activation include miR-17-5p [95, 127], miR-1246 [49, 68], miR-30c [62, 122], and miR-181a [40, 62].

In summary, this body of research indicates that miRNAs which promote activation of HSCs are part of the cargo of circulating exosomes released from PDAC cells. Therefore, and although experimental support is required, we hypothesise that miRNA-carrying exosomes derived from PDAC can be taken up by the liver where they can activate HSCs to increase hepatic fibrosis and facilitate the formation of the hepatic metastatic niche.

## Concluding remarks

Accumulating evidence has proved the key role of miRNAs in the activation of CAFs. Furthermore, circulating exosomal miRNAs have been proposed as predictive and prognostic biomarkers in cancer [41, 42, 58]. In the context of pancreatic cancer, multiple functional analyses have confirmed the significant role that exosomal miRNAs play in tumour cells-CAFs crosstalk, PDAC pathogenesis, diagnosis, and response to therapy [11, 12, 43, 81, 100, 117].

Pancreatic cancer is highly metastatic, with liver the most common site of distant metastasis. This preference for hepatic dissemination is mediated by a selective remodelling of the liver microenvironment to generate a hepatic metastatic niche that promotes and facilitates the engraftment of PDAC cells and the development of metastatic deposits [14]. Activated HSCs increase fibrosis and are major mediators of the formation of the hepatic metastatic niche. Since miRNAs have been shown to contribute to driving the activation of HSCs, research focused on investigating the role of circulating exosomal miRNAs derived from PDAC cells in the activation of HSCs, and generation of a metastatic niche in the liver, is warranted.

Collectively, the findings presented in this review strongly suggest that miRNAs constitute an attractive target for cancer therapy. The development of miRNA-based therapies employing extracellular vesicles loaded with miRNA mimics, for restoring miRNA loss expression, or antagomiRs for inactivating overexpressed miRNAs, may constitute a promising therapeutic tool to generate effective anti-cancer therapies to fight pancreatic cancer.
